# Analysis of global, regional and national trends in the burden of soft tissue and other extraosseous sarcomas from 1990 to 2021: A systematic analysis of the global burden of disease study 2021

**DOI:** 10.1371/journal.pone.0332796

**Published:** 2025-09-19

**Authors:** Xing Chen, Zhixiong Zhang, Jiwei Xiang, Ruliu Xiong, Xingmao Zhou

**Affiliations:** Department of Orthopaedic Surgery, Zhongshan Hospital of Guangzhou University of Traditional Chinese Medicine, Zhongshan, P.R. China; National Center for Chronic and Noncommunicable Disease Control and Prevention, Chinese Center for Disease Control and Prevention, CHINA

## Abstract

**Background:**

Soft tissue and other extraosseous sarcomas (STSES) are rare malignant tumors originating from mesenchymal tissues with complex etiologies. A systematic analysis of global burden trends is urgently needed.

**Methods:**

Utilizing the Global Burden of Disease (GBD) 2021 database, we assessed STSES incidence, mortality, disability-adjusted life years (DALYs), age-standardized incidence rate (ASIR), age-standardized mortality rate (ASMR), and age-standardized DALY rate (ASDR) across regions and countries from 1990 to 2021, stratified by sociodemographic index (SDI). Spatiotemporal models, Joinpoint regression (to calculate average annual percentage changes, AAPC), and decomposition analysis were employed to evaluate the impacts of population growth, aging, and epidemiological factors on disease burden.

**Results:**

In 2021, there were 96,201 new STSES cases globally, resulting in 50,203 deaths and 1.678 million DALYs. While age-standardized rates (per 100,000 person-years) showed declining trends (AAPC for ASIR = −0.13, ASMR = −0.60, ASDR = − 0.94), absolute burden increased by 77.97% due to population growth. Males exhibited consistently higher burden than females, with incidence peaking at 55–74 years. Notably, young females (10–29 years) transiently surpassed males in DALYs. Significant regional disparities emerged: High-SDI regions (e.g., Western Europe) demonstrated lower ASMR attributable to advanced diagnostics and treatment, whereas low-SDI regions (e.g., Uganda, ASMR = 1.96/100,000) faced poorer prognoses due to healthcare deficiencies. SDI exhibited a nonlinear association with disease burden—middle-SDI regions (0.4–0.8) showed rising ASIR, potentially linked to industrial pollution and improved diagnostic capabilities. These findings underscore the critical need for context-specific prevention and resource allocation strategies to address the evolving global STSES burden.

**Conclusions:**

The global STSES burden is predominantly driven by population growth, necessitating targeted prevention strategies addressing occupational exposures in males and subtype-specific risks among young females. While high-SDI regions demonstrate mortality reduction through precision oncology, low-SDI regions require urgent improvements in healthcare accessibility to mitigate survival disparities. Persistent regional heterogeneity underscores the imperative for international collaboration to standardize diagnostic protocols and ensure equitable resource allocation. These insights emphasize the need for stratified surveillance systems and translational research to optimize context-specific intervention frameworks.

## 1. Introduction

Soft tissue sarcomas are a group of malignant tumors originating from non-epithelial mesenchymal tissues that account for approximately 1% of malignant tumors in adults and 15% of malignant tumors in children [1]. These tumors may arise in any anatomic site, with the highest prevalence in the extremities (43%), trunk (10%), viscera (19%), retroperitoneum (15%), and head/neck region (9%). Their clinical presentation is often insidious, initially manifesting as painless masses that may later cause compressive symptoms or functional impairment as they enlarge [2,3].Histologically, soft tissue sarcomas are classified into over 100 subtypes. The most frequent subtypes include liposarcoma (20%), leiomyosarcoma (15%), undifferentiated pleomorphic sarcoma (10%), synovial sarcoma (10%), and malignant peripheral nerve sheath tumors (5%) [4,5]. Extraskeletal sarcomas specifically denote bone- or cartilage-origin sarcomas occurring outside the osseous system, such as extraskeletal osteosarcoma (<5%) and extraskeletal Ewing sarcoma (<1%). Although these tumors share pathological features with their intraosseous counterparts, they exhibit worse prognoses [6,7]. The precise etiology of STSES remains complex and largely unknown, generally attributed to a combination of genetic predisposition, environmental exposures, and random genetic mutations. Specific hereditary syndromes, notably Li-Fraumeni syndrome and Neurofibromatosis type 1, confer significant risk through mutations in key genes such as TP53 and NF1 [8]. Established environmental risks include radiation therapy, which can lead to post-radiation sarcomas; long-term vinyl chloride exposure, strongly linked to hepatic angiosarcoma; chronic severe lymphedema, predisposing to lymphangiosarcoma; and immunosuppression, as seen in organ transplantation or HIV/AIDS, which substantially elevates the risk of Kaposi sarcoma-itself closely associated with HHV-8 infection – and certain leiomyosarcomas. Despite these known associations, the vast majority of STSES cases, exceeding 80%, are idiopathic, lacking identifiable triggers, and are thought to arise from acquired random genetic mutations or other unknown factors [9]. Diagnosis requires multimodal approaches: MRI/CT for local staging, PET-CT for metastatic evaluation, and histopathological biopsy supplemented by immunohistochemistry and molecular testing (e.g., SYT-SSX fusion genes for synovial sarcoma). Treatment paradigms emphasize wide-margin surgical resection, augmented by neoadjuvant/adjuvant radiotherapy and chemotherapy. For advanced disease, targeted therapies (e.g., pazopanib, trabectedin) and immunotherapy (programmed cell death protein 1, PD-1 inhibitors) are increasingly utilized. The overall 5-year survival rate ranges from 50% to 65%, whereas metastatic disease portends a dismal prognosis with a median survival of 12–18 months [3,10,11]. Thus, comprehensive understanding of the disease burden and epidemiological trends of soft tissue and extraskeletal sarcomas is imperative.

In this study, we conducted a systematic assessment of the disease burden of soft tissue and other extraosseous sarcomas (STSES) across 204 countries and territories worldwide, utilizing data from the GBD 2021 database. Using a multidimensional analytical framework, we first established a global prevalence trend model for the period from 1990 to 2021, followed by an analysis of spatial heterogeneity to reveal geographic distribution patterns, and demographic stratification to assess age- and sex-specific disease burden.We incorporated the Socio-demographic Index (SDI), obtained from the official website of the GBD database (https://ghdx.healthdata.org/gbd-2021), into a multivariate regression model to quantitatively evaluate the association between socioeconomic development levels and disease burden.This systematic assessment not only enhances the scientific understanding of STSES epidemiology but also provides an evidence-based foundation for optimizing resource allocation strategies and developing targeted public health interventions through the creation of geographic distribution maps and risk prediction models.

## 2. Methodology

### 2.1 Overview

The GBD 2021 (Global Burden of Disease Study 2021), led by the Institute for Health Metrics and Evaluation (IHME) at the University of Washington, USA, systematically evaluates health losses attributable to diseases across 204 countries and territories [12]. The study employs a standardized methodological framework to quantify population health impacts using disability-adjusted life years (DALYs) as the primary metric. DALYs integrate years of life lost (YLL) and years lived with disability (YLD) (equation: DALYs = YLL + YLD). Here, YLL represents health loss due to premature mortality (calculated as the number of deaths in a population × the difference from standardized life expectancy), while YLD measures health loss from disability (derived from disease prevalence × WHO disability weights) [13].The study integrated heterogeneous data from multiple sources, including global vital registration systems, cause-of-death inference models, epidemiological surveys, health surveillance records, and demographic reports. Parameter estimation employed the following analytical frameworks:Spatiotemporal disease prevalence modeling with cross-population consistency, implemented through the DisMod-MR 2.1 Bayesian meta-regression framework, and mortality calibration using the Cause of Death Ensemble Model (CODEm) to optimize multivariate parameter accuracy [14]. As a core evaluation index of regional development level, the calculation of SDI integrates economic, educational and demographic data in three dimensions: per capita income with a lag of 25 years is used to measure the distribution of economic resources, the average years of education of the population aged 15 years or older is used to characterize human capital, and the total fertility rate is used to reflect the demographic structure, and the geometric means of the three are standardized to map to the interval of 0–100 (0 = lowest development level, 100 = highest development level). The three geometric means were normalized and mapped to the 0–100 range (0 = lowest development level, 100 = highest development level). Based on the SDI thresholds, the study classifies the globe into five levels of development: low (0–0.45), medium-low (0.45–0.61), medium (0.61–0.69), medium-high (0.69–0.81) and high SDI (0.81–1.00) [15]. To ensure robustness, age-standardized rates (per 100,000 population) were calculated using the GBD reference demographic structure. A 95% uncertainty interval (UI) was generated through 500 iterative samplings, with confidence ranges defined at the 2.5th–97.5th percentiles [16]. The analytical workflow adhered strictly to the Institute for Health Metrics and Evaluation (IHME) standardized protocol (GBD Compare) to guarantee methodological reproducibility and cross-regional comparability.

### 2.2 Data sources

The data for this study were obtained from the 2021 public database of the Global Burden of Disease Study (GBD, accessible at https://vizhub.healthdata.org/gbd-results/). This database systematically integrates health assessment data from 204 countries/regions worldwide, covering 369 diseases and injuries over the period from 1990 to 2021. Core indicators extracted include the incidence, death toll, and total disability-adjusted life years (DALYs) of soft tissue and extraosseous sarcomas, along with ASIR, ASMR and ASDR. The study further conducted stratified analyses of disease distribution patterns across age groups, comprehensively collecting age-specific epidemiological parameters. The data architecture employs a three-dimensional spatiotemporal model, longitudinally spanning a 32-year continuous observation period and transversely incorporating multidimensional indicators such as demographic characteristics, geographic regions, and standardized rates. This framework comprehensively delineates the spatiotemporal heterogeneity of disease burden [17,18].

While the GBD study provides invaluable standardized estimates for global comparative analyses, it is crucial to acknowledge inherent limitations associated with its modeling approach, particularly relevant for rare cancers like STSES. A key limitation is the Model Dependency. GBD estimates are derived through complex statistical models (e.g., Dis Mod-MR, CODEm) that synthesize heterogeneous data sources and employ imputation for regions with missing or sparse data [19]. While robust, these estimates represent modeled predictions rather than direct observations everywhere. Another consideration is underestimation in Data-Sparse Regions.The accuracy of GBD estimates is heavily contingent on the quality and coverage of underlying input data [20]. In low-resource settings and regions with underdeveloped vital registration systems or cancer registries, insufficient death certification, lack of pathological confirmation, and limited diagnostic capacity for rare tumors like STSES can lead to significant underestimation of true incidence and mortality. A related challenge is posed by rare subtypes. Extra skeletal sarcomas, being exceptionally rare, are particularly susceptible to underdiagnosis, misclassification, or incomplete reporting even in high-resource settings. This inherent difficulty in ascertainment is amplified in regions with constrained healthcare resources, posing a greater risk of underestimation for these specific entities within the broader STSES category [21]. These limitations were considered when interpreting the results, especially the observed regional disparities.

### 2.3 Statistical analysis

This study analyzed the global epidemiological trends of STSES from 1990 to 2021, encompassing crude incidence, age-standardized incidence rate (ASIR), mortality, age-standardized mortality rate (ASMR), disability-adjusted life years (DALYs), and age-standardized DALY rate (ASDR). The Age-Standardized Rate (ASR) is a core metric used to compare disease frequency across populations or over time while eliminating the impact of age-structure differences. Its calculation formula is: ASR = [Σ (aᵢ × wᵢ)/ Σ wᵢ} × 100,000 [22]. Maps illustrating the 2021 burden of STSES were generated to highlight global geographic variations, and the age-specific burden distribution was also presented. To quantify trends, Joinpoint regression analysis was performed using Joinpoint Regression Software (v4.5.0, National Cancer Institute). The grid search method identified trend turning points, with model selection based on the Bayesian Information Criterion (BIC) (maximum joinpoints = 5). We employed the best-fitting model, expressing the slope of each segment between joinpoints as the APC and the AAPC [23]. Monte Carlo permutation tests with 500 iterations established 95% confidence intervals for trend segments. An AAPC > 0 indicates an upward trend, < 0 a downward trend, and = 0 a stable trend [24]. Das Gupta’s calculation method was used, which decomposes the overall change into multiple parts through mathematical decomposition techniques to identify the specific contributions of each part. This is also a commonly used method in decomposition analysis. The calculation formula is ${IC}_{ay,py,ey}=\sum_{i=1}^{20}\left(a_{i,y}.p_{y}.e_{i,y}\right)$ [25]. We applied decomposition analysis to examine the contribution of aging, population growth and epidemiological changes to incidence rate, mortality and DALYs trends between 1990 and 2021. For all GBD regions, we provided survey results based on corresponding SDI values and employed Spearman’s rank correlation analysis to assess the association between age-standardized rates and SDI across 21 regions [26]. We used R statistical software (version 4.3.2) and Joinpoint (version 4.5.0) to analyze and visualize the data.

## 3. Results

### 3.1 Global burden of soft tissue and other extraosseous sarcomas

**Fig 1:**
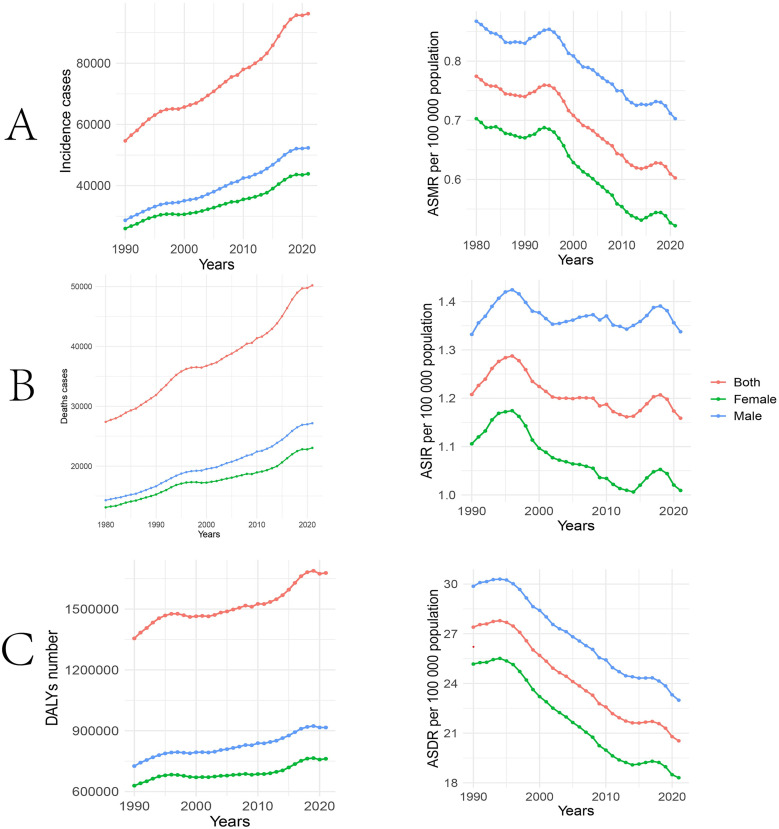
Temporal trends of Soft tissue and other extraosseous sarcomas in global from 1990 to 2021. **A:** The time trend of global soft tissue and other extra bone sarcoma incidence cases and ASIR. **B:** The time trend of global soft tissue and other extra bone sarcoma deaths cases and ASMR. **C:** The time trend of global soft tissue and other extra bone sarcoma DALYs numbe and ASDR. ASIR, age-standardized incidence rate; ASMR, age-standardized deaths rate; ASDR, age-standardized DALYs rate; DALYs,Disability-Adjusted Life Years.

It is unsurprising that both genders showed a continuous climb in all three dimensions of incidence, number of deaths, and disability-adjusted life years (DALYs) between 1990 and 2021, this likely reflects population growth. But it is noteworthy that the burden of disease was significantly higher in the male group than in the female group. The ASIR peaked in 2006, with a small peak in 2014, but stabilized overall. Male prevalence is higher than both genders and females.ASMR and ASDR show an overall decreasing trend (Fig 1).In 2021, the global number of STSES morbidity is 96201 (83,424–116,185) cases, deaths are 50,203 (43,232–61,280) cases, and the global DALYs for STSES in 2021 were 16,778,792 (95% CI: 14,282,209–21,157,070) years.From 1990 to 2021, the AAPC for the ASIR was −0.13 (95% UI: −0.22 to 0.04). Similarly, the AAPC for the ASMR was −0.60 (95% UI: −0.67 to 0.53), and for the ASDR was −0.94 (95% UI: −1.02 to 0.85) (Table 1).

**Table 1:** Incidence rate, age standardized incidence rate, number of deaths, age standardized mortality and age standardized disability adjusted life year rate of Soft tissue and other extraosseous sarcomas in 1990 and 2021.

Location	Number_1990	Number_2021	Rate_1990	Rate_2021	AAPC
	(95%UI)	(95% UI)	(95% UI)	(95% UI)	(95% UI)
**Incidence**					
Global	54631 (46757,64000)	96201 (83424,116185)	1.21 (1.04,1.39)	1.16 (1,1.41)	−0.13 (−0.22,0.04)
High SDI	18831 (17963,19524)	34515 (31573,36742)	1.86 (1.78,1.93)	2.05 (1.9,2.16)	
High-middle SDI	10195 (8744,11367)	17875 (15748,20645)	1.01 (0.87,1.12)	1.02 (0.9,1.18)	
Middle SDI	10281 (7831,12758)	19510 (15577,24983)	0.77 (0.58,0.94)	0.75 (0.6,0.97)	
Low-middle SDI	8738 (6305,11299)	14332 (11177,20217)	0.96 (0.68,1.22)	0.88 (0.69,1.24)	
Low SDI	6528 (4992,9604)	9870 (7513,14891)	1.58 (1.22,2.26)	1.25 (0.97,1.87)	
Andean Latin America	361 (249,460)	647 (499,824)	1.2 (0.84,1.51)	1.04 (0.81,1.33)	
Australasia	472 (435,508)	1127 (981,1283)	2.11 (1.94,2.27)	2.52 (2.18,2.88)	
Caribbean	480 (408,572)	691 (552,836)	1.54 (1.33,1.81)	1.39 (1.1,1.71)	
Central Asia	347 (292,416)	798 (663,943)	0.61 (0.52,0.74)	0.91 (0.76,1.08)	
Central Europe	1564 (1421,1712)	2980 (2666,3282)	1.15 (1.05,1.26)	1.68 (1.51,1.86)	
Central Latin America	1221 (1130,1325)	3441 (3097,3803)	0.97 (0.9,1.05)	1.34 (1.21,1.49)	
Central Sub-Saharan Africa	493 (310,790)	913 (602,1445)	1.08 (0.7,1.69)	1 (0.66,1.6)	
East Asia	6169 (4412,7945)	9865 (6953,13828)	0.65 (0.46,0.83)	0.5 (0.35,0.7)	
Eastern Europe	2836 (2359,3144)	4195 (3758,4626)	1.11 (0.93,1.24)	1.44 (1.28,1.58)	
Eastern Sub-Saharan Africa	3653 (2682,5651)	5557 (3916,8748)	2.53 (1.93,3.82)	2.04 (1.48,3.2)	
High-income Asia Pacific	1984 (1845,2198)	3884 (3368,4346)	1.07 (0.99,1.19)	1.17 (1.03,1.29)	
High-income North America	8590 (8185,8941)	14069 (13059,14825)	2.68 (2.57,2.78)	2.63 (2.48,2.77)	
North Africa and Middle East	2943 (2198,4071)	4366 (3269,6211)	1.14 (0.81,1.51)	0.84 (0.63,1.18)	
Oceania	11 (8,16)	21 (14,30)	0.26 (0.19,0.35)	0.2 (0.14,0.28)	
South Asia	8533 (5501,10592)	13552 (10001,19135)	1.02 (0.65,1.26)	0.85 (0.63,1.21)	
Southeast Asia	2702 (1992,3692)	4787 (3710,6797)	0.75 (0.55,1.02)	0.71 (0.55,1.01)	
Southern Latin America	615 (542,694)	1097 (948,1234)	1.3 (1.14,1.46)	1.39 (1.19,1.57)	
Southern Sub-Saharan Africa	364 (257,475)	847 (560,1083)	0.9 (0.64,1.17)	1.22 (0.8,1.55)	
Tropical Latin America	1080 (987,1189)	3070 (2842,3308)	0.9 (0.82,0.98)	1.24 (1.14,1.34)	
Western Europe	8637 (8158,9052)	17398 (15679,18764)	1.78 (1.68,1.86)	2.41 (2.21,2.59)	
Western Sub-Saharan Africa	1577 (1102,2623)	2896 (1995,4519)	0.86 (0.61,1.44)	0.79 (0.57,1.2)	
location	Number_1990	Number_2021	Rate_1990	Rate_2021	
	(95%UI)	(95% UI)	(95% UI)	(95% UI)	
**Deaths**					
Global	31878 (26446,37708)	50203 (43232,61280)	0.74 (0.62,0.86)	0.6 (0.52,0.74)	−0.60 (−0.67,0.53)
High SDI	8945 (8576,9230)	15149 (13950,15973)	0.86 (0.82,0.88)	0.81 (0.76,0.85)	
High-middle SDI	5756 (4891,6435)	8569 (7525,9840)	0.58 (0.5,0.65)	0.47 (0.41,0.54)	
Middle SDI	6504 (4864,8044)	10866 (8705,13645)	0.55 (0.41,0.67)	0.42 (0.34,0.53)	
Low-middle SDI	6068 (4313,7677)	9245 (7218,12795)	0.77 (0.54,0.96)	0.61 (0.48,0.84)	
Low SDI	4571 (3586,6636)	6322 (4882,9481)	1.33 (1.02,1.87)	0.95 (0.75,1.42)	
Andean Latin America	236 (168,292)	372 (287,473)	0.9 (0.64,1.12)	0.61 (0.48,0.78)	
Australasia	216 (201,231)	459 (397,521)	0.95 (0.88,1.01)	0.94 (0.82,1.08)	
Caribbean	291 (249,339)	393 (312,477)	0.98 (0.85,1.13)	0.78 (0.61,0.96)	
Central Asia	210 (178,252)	442 (367,520)	0.4 (0.34,0.48)	0.53 (0.44,0.62)	
Central Europe	895 (819,971)	1480 (1338,1630)	0.65 (0.6,0.71)	0.76 (0.69,0.84)	
Central Latin America	723 (690,752)	1875 (1686,2069)	0.66 (0.63,0.69)	0.74 (0.67,0.82)	
Central Sub-Saharan Africa	336 (226,534)	595 (390,940)	0.89 (0.59,1.4)	0.77 (0.49,1.26)	
East Asia	3981 (2798,5098)	4963 (3519,6883)	0.46 (0.32,0.58)	0.25 (0.18,0.34)	
Eastern Europe	1592 (1341,1750)	2144 (1919,2357)	0.61 (0.52,0.67)	0.68 (0.61,0.75)	
Eastern Sub-Saharan Africa	2576 (1955,3900)	3580 (2583,5630)	2.17 (1.7,3.23)	1.58 (1.18,2.45)	
High-income Asia Pacific	887 (825,988)	1710 (1473,1894)	0.47 (0.44,0.53)	0.43 (0.38,0.47)	
High-income North America	3906 (3735,4018)	6196 (5732,6470)	1.17 (1.13,1.21)	1.07 (1,1.11)	
North Africa and Middle East	1752 (1286,2322)	2235 (1644,3108)	0.79 (0.55,1)	0.46 (0.34,0.63)	
Oceania	8 (6,10)	13 (9,19)	0.2 (0.14,0.27)	0.14 (0.1,0.2)	
South Asia	6073 (3875,7392)	8932 (6603,12593)	0.84 (0.53,1.02)	0.59 (0.44,0.83)	
Southeast Asia	1744 (1263,2377)	2873 (2252,4047)	0.55 (0.4,0.75)	0.44 (0.35,0.62)	
Southern Latin America	373 (332,418)	571 (499,642)	0.8 (0.71,0.9)	0.69 (0.6,0.78)	
Southern Sub-Saharan Africa	223 (158,289)	524 (356,661)	0.62 (0.44,0.81)	0.8 (0.54,1.01)	
Tropical Latin America	667 (628,706)	1721 (1592,1831)	0.61 (0.57,0.65)	0.69 (0.63,0.73)	
Western Europe	4184 (3949,4377)	7437 (6709,7969)	0.81 (0.76,0.84)	0.91 (0.83,0.97)	
Western Sub-Saharan Africa	1005 (724,1653)	1687 (1190,2534)	0.67 (0.49,1.08)	0.57 (0.42,0.86)	
location	Number_1990	Number_2021	Rate_1990	Rate_2021	
	(95%UI)	(95% UI)	(95% UI)	(95% UI)	
**DALYs**					
Global	1355268 (1117320,1670807)	1677892 (1428209,2115701)	27.4 (22.61,33.29)	20.54 (17.46,26.09)	−0.94 (−1.02,0.85)
High SDI	289921 (280450,298395)	402671 (378751,420655)	29.85 (28.95,30.76)	27.03 (25.54,28.19)	
High-middle SDI	212759 (181387,240752)	244695 (215778,282509)	20.56 (17.54,23.25)	14.86 (13.1,17.14)	
Middle SDI	292240 (222251,364036)	363071 (292600,457258)	19.6 (14.77,24.28)	13.93 (11.21,17.61)	
Low-middle SDI	301445 (224549,389331)	357408 (280076,502830)	28.98 (20.77,36.7)	20.64 (16.15,28.91)	
Low SDI	257544 (200596,379547)	308353 (232677,465079)	52.72 (41.34,76.24)	33.41 (25.63,50.17)	
Andean Latin America	11335 (7935,14357)	12639 (9628,16280)	33.65 (23.86,41.8)	19.89 (15.18,25.59)	
Australasia	6789 (6285,7282)	12418 (10813,14176)	30.96 (28.58,33.21)	30.81 (26.44,35.31)	
Caribbean	13634 (10863,16877)	16016 (12029,20782)	40.19 (32.89,48.48)	33.74 (24.83,44.29)	
Central Asia	8655 (7236,10508)	15806 (13045,18739)	14.3 (12.09,17.25)	17.18 (14.21,20.34)	
Central Europe	30745 (28305,33648)	39745 (35838,43808)	23.11 (21.26,25.33)	25.08 (22.55,27.66)	
Central Latin America	34832 (33345,36424)	67919 (60880,75101)	24.52 (23.44,25.54)	26.25 (23.53,29.07)	
Central Sub-Saharan Africa	19908 (12777,31780)	29079 (18912,45073)	36.66 (24.64,57.87)	27.6 (18.14,43.63)	
East Asia	158637 (113892,205561)	133309 (94782,187769)	15.18 (10.81,19.61)	6.93 (4.93,9.81)	
Eastern Europe	55230 (46085,60859)	63705 (57292,70235)	22.22 (18.7,24.42)	23.34 (20.87,25.78)	
Eastern Sub-Saharan Africa	144947 (106859,221263)	171955 (121266,272847)	83.02 (63.67,124.2)	53.14 (38.49,83.55)	
High-income Asia Pacific	33552 (30987,38073)	40354 (35482,44359)	18.48 (16.98,21.05)	15.2 (13.37,16.55)	
High-income North America	127490 (123682,130700)	173363 (164934,180053)	41.34 (40.25,42.34)	35.69 (34.1,37)	
North Africa and Middle East	87427 (67030,121517)	87246 (66215,123402)	29.53 (21.89,39.54)	15.42 (11.57,21.7)	
Oceania	353 (255,481)	597 (416,869)	7 (5.15,9.56)	5.11 (3.55,7.24)	
South Asia	293413 (197559,358163)	326478 (245107,462756)	30.71 (19.81,37.5)	19.33 (14.53,27.35)	
Southeast Asia	82667 (61429,113150)	103177 (80571,146440)	20.53 (14.96,28.14)	14.69 (11.5,20.97)	
Southern Latin America	13672 (12021,15427)	18244 (15817,20677)	28.36 (24.94,31.98)	24.11 (20.82,27.52)	
Southern Sub-Saharan Africa	10914 (7620,14045)	21910 (14853,28122)	24.44 (17.25,31.6)	29.53 (20.09,37.68)	
Tropical Latin America	29791 (28061,31772)	60953 (56779,64974)	22.64 (21.31,24.05)	24.85 (23.08,26.59)	
Western Europe	128507 (122201,134346)	188795 (174125,201378)	28.44 (27.13,29.7)	30.34 (28.15,32.31)	
Western Sub-Saharan Africa	62771 (43766,103396)	94187 (64643,147590)	28.58 (20.76,46.91)	20.92 (14.83,31.34)	

### 3.2 Overview of the regional, and national burden, 1990–2021

**Fig 2:**
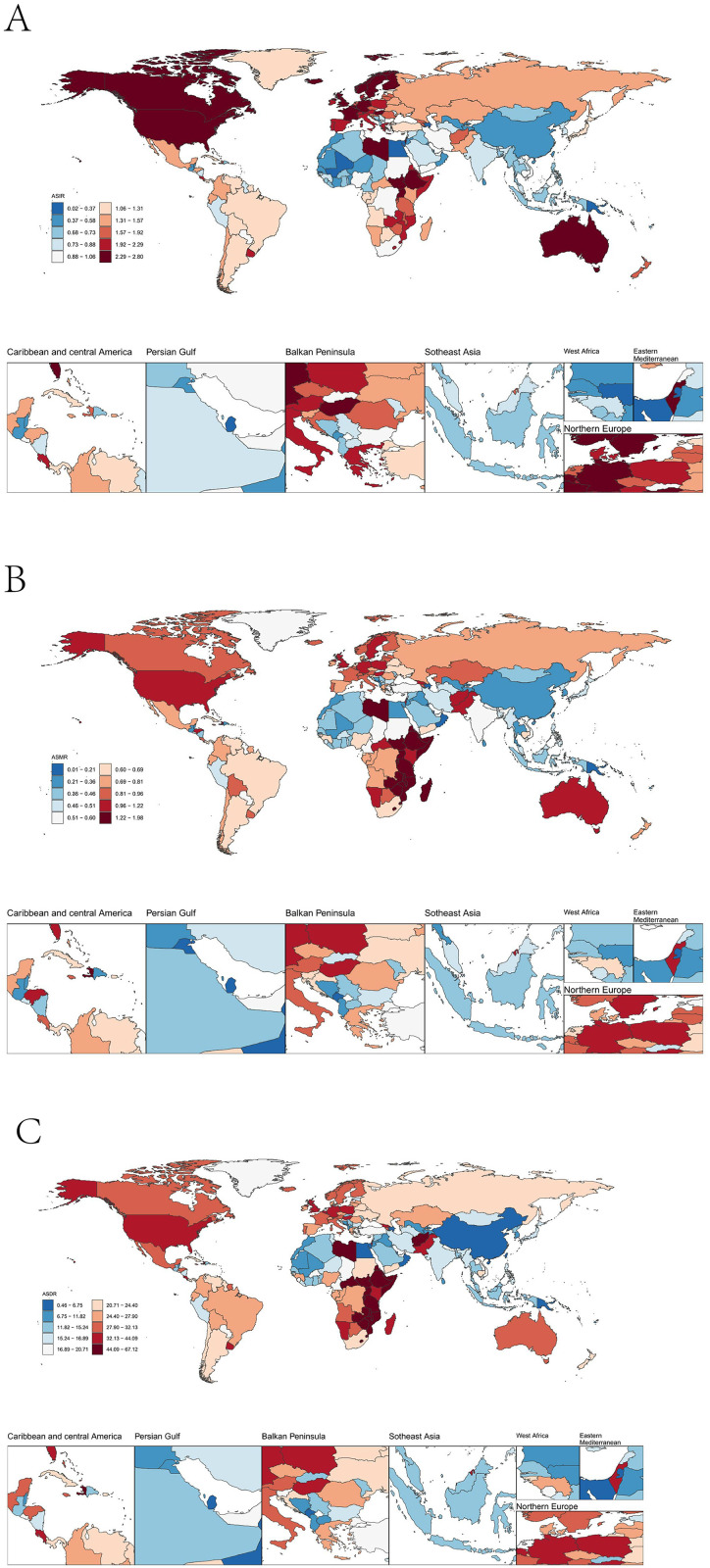
Global distribution of soft tissue and other extraosseous sarcomas in 2021. **A:** Global distribution of ASIR in soft tissue and other extra bone sarcomas. **B:** Global distribution of ASMR in soft tissue and other extra bone sarcomas. **C:** Global distribution of ASDR in soft tissue and other extra bone sarcomas. ASIR, Age standardized incidence rate; ASMR, Age standardized mortality rate; ASDR, Age standardized DALYs rate.

In 2021, substantial healthcare inequities manifested in STSES burden disparities: Twenty-three countries reported zero incident cases while an additional four documented no deaths(Additional file1). Notably, high-SDI nations such as Germany exhibited the highest ASIR at 2.8 per 100,000 population, potentially indicating overdiagnosis or industrial exposures. Conversely, low-SDI regions exemplified by Uganda showed the highest ASMR of 1.96 per 100,000, suggesting critical gaps in diagnosis and therapy. Although populous countries including the USA and India carried the largest absolute burden due to demographic scale, their age-standardized rates remained lower than those in high-SDI areas. (Fig 2, Table 1).

### 3.3 Age distribution of burden

**Fig 3:**
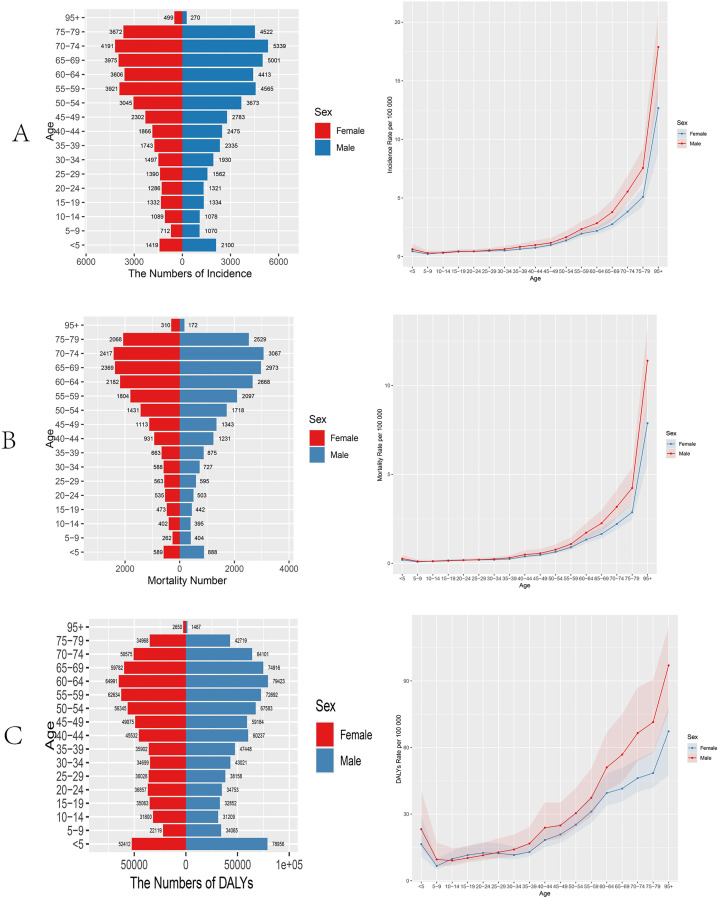
Age distribution of soft tissue and other extraosseous sarcomas burden by gender in 2021. **A:** Age distribution of incidence rate and number of incidence burden for soft tissue and other extra bone sarcomas by gender. **B:** Age distribution of mortality and mortality numbe burden for soft tissue and other extra bone sarcomas by gender. **C:** Age distribution of DALYs rate and number of DALYs burden for soft tissue and other extra bone sarcomas by gender. DALYs:Disability adjusted life years.

The age distribution shows significant differences. Morbidity rates were similar for males and females between ages 14–29, but were higher in males in other age groups. The population-wide number of morbidities declined briefly in the initial phase up to the 5–9 age group, then continued to rise until the 55–59 age group; the number of morbidities declined briefly in the 60–64 age group, peaked in the 70–74 age group, and then continued to decline (Fig 3A). Mortality trends were generally consistent with morbidity. Sex differences were particularly striking, with males having higher deaths than females at all ages except the 95 + age group, and growth in both sexes from the 5–9 age group to the 70–74 age group showing a continuous upward trend, followed by a continuous decline to a minimum (Fig 3B).The rate curve for disability-adjusted life years (DALYs) shows a different distribution for females in the 10–29 age group than for males, and for males in the rest of the age group than for females; the curve for the population as a whole continues to move upward after a downward trend in the 5–9 year age group, and then briefly declines for females in the 20–34 year age group, with the slope of the increase for males being slightly higher than that for females. 9 years old) briefly downward, the second stage (10–14 years old to 20–24 years old) rebounded and then declined again, and the third stage (25–29 years old to 60–64 years old) steadily climbed to the peak and then declined. (Fig 3C).

### 3.4 Joinpoint regression analysis of trends in soft tissue and other extraosseous sarcomas

**Fig 4:**
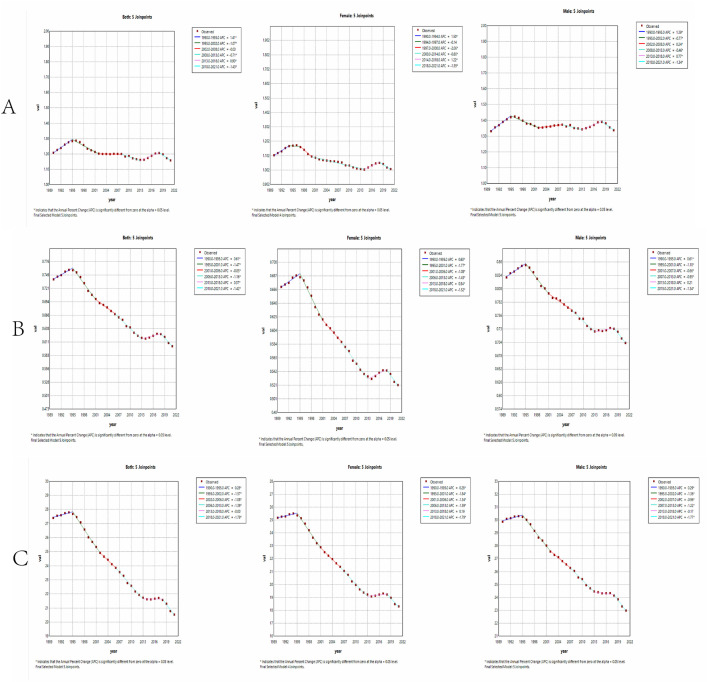
Joinpoint regression analysis of young soft tissue and other extraosseous sarcomas from 1990 to 2021. **A:** Joint point regression analysis of ASIR in soft tissue and other osteosarcoma. **B:** Joint point regression analysis of ASMR in soft tissue and other osteosarcoma. **C:** Joint point regression analysis of ASDR in soft tissue and other osteosarcoma. ASIR, Age standardized incidence rate; ASMR, Age standardized mortality rate; ASDR, Age standardized DALYs rate.

For the ASIR of STSES, the AAPC from 1990 to 2021 was −0.13 (−0.22,0.04), with an increasing trend globally from 1990 to 1995, then flat from 1995 to 2002, 2002–2008, a slight decrease from 2008 to 2013, and an increasing trend from 2013 to 2018; 2018–2021 showed the most significant decline with an Annual Percentage Change (APC) of −1.43 (−1.89, 0.96). There is a total of 5 join points. Age-standardized incidence rates (ASIR) showed similar temporal trends for both sexes. Globally, trends in ASMR and ASDR were also consistent, with parallel patterns observed in males and females. Globally, the AAPC for ASMR from 1990 to 2021 was −0.60 (−0.67,0.53), ASMR trended upward from 1990 to 1995, and continued to decline from 1995 to 2013, with the largest decline from 1995 to 2001, with an AAPC of 1.47 (−1.64, 1.30); from 2013 to 2018 a smaller increase and then a continuous decline from 2018 to 2021. In total, it has experienced five connection points. Globally, the AAPC for ASDR from 1990 to 2021 is −0.94 (−1.02, 0.85), with ASDR rising from 1990 to 1995, and declining in all subsequent periods except for 2013–2018 when it remained flat, with the fastest decline occurring from 2018 to 2021, with an APC of 1.79 (−2.20, 1.37), with five connections during this period (Fig 4, Table 1).

### 3.5 Decomposition analysis of incidence rate, mortality and DALYs

**Fig 5:**
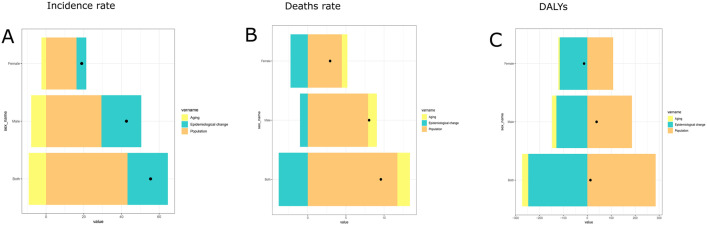
Decomposition analysis of global incidence, mortality, and DALYs trends (1990-2021) for soft tissue and other extraskeletal sarcomas. **A:** Decomposition analysis of global incidence trends for soft tissue and other extraskeletal sarcomas; **B:** Decomposition analysis of global death tends for soft tissue and other extraskeletal sarcomas.; **C:** Decomposition analysis of global DALYs tends for soft tissue and other extraskeletal sarcomas.DALYs:Disability adjusted life years. The black dots represent the overall change values caused by all three reasons.

Analysis of global factors from 1990 to 2021 revealed their distinct impacts on STSES burden: aging (−16.54%), population growth (77.97%), and epidemiological changes (38.56%) on incidence; aging (17.12%), population growth (122.64%), epidemiological changes (−39.78%) on mortality; aging (−195.55%), population growth (2198.28%), epidemiological changes (−1902.73%) on DALYs. Crucially, population growth was the primary driver of increased burden, significantly elevating rates of incidence, mortality, and DALYs. (Fig 5, Table 2)

**Table 2:** Incidence rate, mortality and DALYs of global soft tissue and other extraosseous sarcomas from 1990 to 2021.

	sex_name	overll_difference	a_effect	p_effect	r_effect	a_percent	p_percent	r_percent	val_1990	val_2021	diff1
**Incidence**											
1	Both	55.45	−9.17	43.234	21.382	−16.54	77.97	38.56	54630.92	96200.96	41570.04
2	Male	42.64	−7.866	29.393	21.113	−18.45	68.93	49.51	28657.36	52347.91	23690.55
3	Female	18.91	−2.409	16.158	5.159	−12.74	85.45	27.28	25973.56	43853.05	17879.49
**Death**											
1	Both	9.58	1.64	11.749	−3.811	17.12	122.64	−39.78	31878.28	50203.14	18324.857
2	Male	8.04	1.149	7.907	−1.017	14.29	98.35	−12.65	16624.89	27162.94	10538.042
3	Female	2.93	0.722	4.465	−2.261	24.64	152.39	−77.17	15253.39	23040.2	7786.815
**DALY**											
1	Both	12.98	−25.383	285.337	−246.974	−195.55	2198.28	−1902.73	1355268.1	1677891.9	322623.8
2	Male	38.79	−18.484	186.467	−129.19	−47.65	480.71	−333.05	725914.5	916038.1	190123.6
3	Female	−13.56	−6.242	107.776	−115.097	46.03	−794.81	848.8	629353.6	761853.8	132500.3

Absolute effect: a_effect、p_effect、r_effect; Relative contribution percentage: a_percent, p_percent, r_percent.

“a-percent” refers to the percentage attributable to population aging, “p-percent” refers to the percentage attributable to population growth, and “r-percent” refers to the percentage attributable to epidemiological factors.

### 3.7 Relationship with SDI

**Fig 6:**
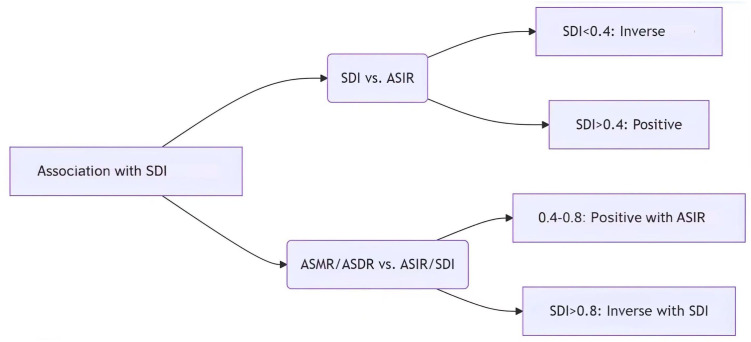
Nonlinear relationships between socio-demographic index and age-standardized rates of soft tissue and extraosseous sarcomas.

**Fig 7:**
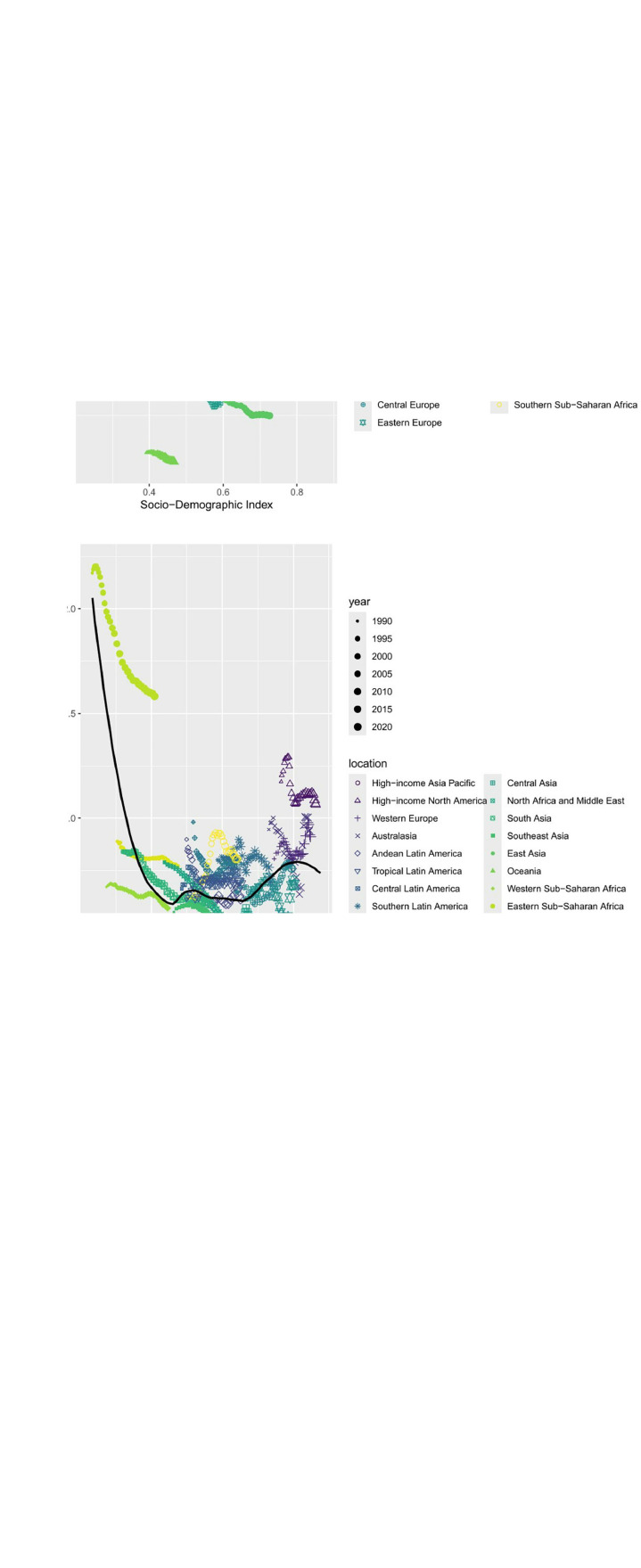
Age standardized rate of soft tissue and other extraosseous sarcomas calculated by sociodemographic index in 21 GBD regions from 1990 to 2021. **A:** ASIR of soft tissue and other extraosseous sarcomas calculated by sociodemographic index in 21 GBD regions. **B:** ASMR of soft tissue and other extraosseous sarcomas calculated by sociodemographic index in 21 GBD regions. **C:** ASDR of soft tissue and other extraosseous sarcomas calculated by sociodemographic index in 21 GBD regions. ASIR, Age standardized incidence rate; ASMR, Age standardized mortality rate; ASDR, Age standardized DALYs rate.

This study systematically analyzes the characteristics of the association between the sociodemographic index (SDI) of STSES and the ASIR、ASMR、 and the ASDR based on data from 1990 to 2021 from 21 regions classified by the Global Burden of Disease Study (GBD). In general, the three indicators showed nonlinear associations with SDI. However, the results showed that in the low SDI interval (SDI < 0.4), ASIR was negatively linearly associated with SDI, with significantly lower values at higher SDI levels; and when the SDI exceeded the 0.4 threshold, the association reversed direction and changed to show a significant positive association. When the SDI was 0.4 to 0.8, ASMR and ASDR were positively associated with ASIR, with larger increases observed in areas with higher SDI levels; when the SDI was greater than 0.8, ASMR and ASDR showed a decreasing decreasing association with SDI (Fig 6). Western Europe, Central Latin America, Caribbean and Eastern SubSaharan Africa have higher-than-expected ASIRs; Southeast Asia, East Asia, Oceania, Central Asia, and High income Asia Pacific have lower-than-expected ASIRs. eastern Sub-Saharan ASMR is higher than expected for Eastern Sub-Saharan Africa, Southern Latin America, Central Latin America, Western Europe, and High-income North America, while ASMR is lower than expected for Southeast Asia, East Asia Oceania, Western Sub-Saharan Africa, Central Asia, and High-income Asia Pacific have lower than expected ASMRs. eastern Sub- Saharan Africa, Central Latin America, Southern Latin America, and High-income North America had higher-than-expected ASDR, while Oceania, Southeast Asia, Central Asia, and High-income Asia Pacific have lower than expected ASDR. (Fig 7)

## 4. Discussion

Drivers and Heterogeneity of Global Burden Trends: The study showed that the overall ASIR of STSES stabilized from 1990 to 2021 (AAPC = −0.13), but the incidence, deaths, and DALYs increased significantly due to population growth. Decomposition analysis revealed that population growth was the predominant driver of this rise, contributing 77.97% to incidence, 122.64% to mortality, and 2198.28% to DALYs. Conversely, aging showed mixed effects—reducing incidence (−16.54%) but increasing mortality (17.12%)—while epidemiological changes contributed positively to incidence (38.56%) but substantially reduced mortality (−39.78%) and DALYs (−1902.73%). It is noteworthy that despite the inverse linear associations in ASMR and ASDR (AAPC = −0.60 and −0.94), the burden of mortality and disability remained significantly higher in low SDI regions than in high SDI regions. This established health inequity aligns with fundamental socioeconomic determinants of health outcomes. The disparity arises from differential access to healthcare resources: high-SDI regions benefit from early diagnosis capabilities and advanced therapeutics, whereas low-SDI regions face systemic barriers including limited healthcare infrastructure, delayed presentation, and constrained treatment availability [27,28]. For example, the extreme difference between Uganda (ASMR = 1.96) and the Northern Mariana Islands (ASMR = 0.01) highlights the critical impact of healthcare accessibility on survival outcomes.

Potential Mechanisms for Differences in Sex and Age Distribution: There are significant sex differences in STSES, with men having higher morbidity, mortality, and DALYs than women. In addition to biological factors (e.g., hormone level differences), occupational exposures (e.g., exposure to chemical carcinogens) and lifestyle (e.g., smoking, alcohol consumption) may further exacerbate the risk in men [29]. Incidence rates peaked among individuals aged 55–74, which is consistent with the age pattern of most solid tumors, suggesting the role of cumulative environmental exposures and decreased DNA repair capacity [30]. However, the rate of DALYs is transiently higher in females than in males in younger age groups (10–29 years), which may be associated with pregnancy-related hormonal changes or gender preference for specific subtypes, such as synovial sarcoma [31]. In addition, the low incidence in children (0–9 years) may reflect a lower association of STSES with genetic syndromes, such as Li-Fraumeni syndrome, or may be related to inadequate early identification of soft tissue masses by pediatric tumor screening systems [32,33].

Non-linear association between socioeconomic development level and disease burden: disease burden in STSES showed a non-linear relationship with SDI: ASIR was inversely associated with development level in low SDI areas(<0.4) areas, which may be associated with infectious disease control, improved malnutrition, and universal access to primary healthcare; whereas, the higher ASIR in medium and high SDI areas (0.4–0.8) may reflect improved diagnostic techniques and increased exposure to environmental risk factors (e.g., industrialized pollution) [34,35]. The stabilizing or decreasing trend of ASIR in high SDI (>0.8) regions may be associated with the use of targeted therapies and immune checkpoint inhibitors, both of which can improve the quality of survival of patients with advanced disease [36,37]. However, the high ASMR and ASDR in low SDI regions suggests that simply improving diagnostic capacity is not enough to improve prognosis and that simultaneous enhancement of treatment accessibility is needed. For example, sub-Saharan Africa, despite low ASIR, has high mortality rates due to insufficient coverage of radiotherapy and chemotherapy, highlighting the serious challenge of unequal resource allocation [38,39].Decomposition analysis identifies population growth as the predominant driver of STSES incidence, accounting for 77.97% of cases. This contribution surpasses that reported for other malignancies like lung cancer [40]. The nonlinear ASIR-SDI relationship, with incidence peaking in middle-SDI regions, parallels patterns seen in industrial pollution-associated cancers such as mesothelioma [41], indicating shared environmental etiology. However, to explain the relationship between ASR and SDI from a single perspective is inadequate. Differences in economic levels, healthcare policies, and cultural factors among countries may also be influencing factors **[42]**. For example, compared to countries with very SDI, those with medium SDI likely have better reporting practices and surveillance systems. Owing to more comprehensive data collection, the reported ASIR and ASMR tend to be higher in these countries, rather than necessarily reflecting an actual increase in disease burden.

Causes of regional and country-level heterogeneity: Regional analyses suggest that Western Europe and high-income North America may have the highest incidence rates globally due to aging populations, advanced diagnostic technologies, and well-established registries. Large populated countries such as India and China, which have a large base and a high burden of deaths and DALYs, have lower age standardization rates than high SDI countries, suggesting a “demographic dividend” effect on the burden [43,44]. Notably, South Asia has the highest burden of DALYs (326,478), which may be associated with delayed diagnosis, unstandardized treatment, and abandonment of treatment due to financial constraints. In addition, some high SDI regions (e.g., Germany) have abnormally high ASIR (2.8/100,000), which requires vigilance for the influence of overdiagnosis or geographically specific risk factors (e.g., industrial chemical exposure) [45,46].

Our findings corroborate recent systematic analyses of STSES burden based on Global Burden of Disease (GBD) 2021 data. Consistent with prior studies [47,48], we observed a 77.97% rise in absolute global incidence—primarily attributable to population growth—while the ASMR declined at the AAPC of −0.60%. Regional mortality disparities (e.g., Uganda and high-SDI nations) further align with reports by Zhu et al. [49]. Uniquely, our decomposition analysis quantified burden drivers: population growth accounted for 77.97%–2198.28% of increases, aging exerted bidirectional effects (−16.54% to 17.12%), and epidemiological changes reduced burdens by up to 1902.73%. We also identified a transient reversal in DALY rates among women aged 10–29 (potentially linked to subtype-specific risks) and established nonlinear SDI associations, including elevated the ASIR in medium-SDI regions. These advances inform stratified resource allocation.

Socioeconomic burden and mitigation strategies: The economic burden of STSES encompasses direct costs, such as wide-margin resection, radiotherapy, and targeted therapies, alongside indirect costs arising from productivity loss due to premature mortality and disability, particularly impacting peak working-age groups aged 55–74 years [50]. Social burdens include caregiver strain, psychological trauma, and the diversion of limited healthcare resources in low-resource settings. Treatment abandonment in South Asia due to financial toxicity exemplifies this latter burden [51]. To mitigate these burdens: High-SDI regions should prioritize cost-effective precision oncology, including PD-1 inhibitors, to reduce relapse-associated expenses. Low-SDI regions urgently require subsidized radiotherapy access and transnational referral networks; regional hub-and-spoke cancer centers in sub-Saharan Africa serve as a model. Gender/age-specific interventions are critical: enhanced surveillance for synovial sarcoma in young females aged 10–29 years and occupational hazard regulations for males exposed to industrial carcinogens. Global standardization of diagnostic protocols via WHO frameworks could curb overdiagnosis-driven burdens, as evidenced by Germany’s elevated ASIR, and align resource allocation with epidemiological needs.

This study has the following limitations: first, GBD data relies on model estimation, and insufficient death registration and pathologically confirmed diagnosis in low-resource countries may lead to underestimation, especially extraosseous sarcoma is more likely to be underdiagnosed due to its rarity; second, STSES contains more than 50 subtypes, which have significant variations in etiology and prognosis, but this study did not stratify subtypes, which may mask key epidemiological features; third, SDI as a composite indicator, fails to fully capture the impact of microfactors such as healthcare systems and cultural practices on disease burden; finally, the impact of the COVID-19 pandemic on the 2020–2021 diagnostic and treatment system was not analyzed separately, which may bias the interpretation of recent trends.

## 5. Conclusion

This study systematically revealed the global disease burden and trends of STSES from 1990–2021. The results showed that STSES incidence, deaths, and DALYs continued to rise due to population growth, with 96,201 incidence cases, 50,203 deaths, and 1.67 million DALYs globally in 2021, with population growth being the main driver (77.97% contribution). Despite the declining trend in ASIR, ASMR, and ASDR, there are significant regional differences: high SDI regions may have lower mortality rates due to advances in diagnostic technology and widespread availability of precision treatment; low SDI regions may have high ASMR and ASDR due to lack of medical resources. Gender differences are prominent, with men having a higher burden than women across the board, and the peak incidence is concentrated in the 55–74 years of age, but the rate of DALYs in young women briefly reverses, suggesting the need for targeted prevention and control. Regional heterogeneity is evident, with overdiagnosis and environmental exposure in Western Europe and North America, and access to treatment in South Asia and sub-Saharan Africa a priority. The study provides key evidence for optimizing global prevention and control strategies, and highlights the need to reduce the burden of STSES disease through equitable distribution of resources, enhanced early screening, and international collaboration.
